# Investigating the Diagnostic and Therapeutic Potential of a T Cell Receptor (TCR)-like single Domain Antibody (sDAb)-Human IgG1 Antibody against Heat Shock Protein (HSP) 16KDa/HLA-A2 for Latent Tuberculosis

**DOI:** 10.3390/tropicalmed9070139

**Published:** 2024-06-26

**Authors:** Huaqiang Liu, Sylvia Annabel Dass, Matthew Tze Jian Wong, Venugopal Balakrishnan, Fazlina Nordin, Gee Jun Tye

**Affiliations:** 1 Institute for Research in Molecular Medicine, University Sains Malaysia, Minden 11800, Malaysia; liuhuaqiang@student.usm.my (H.L.); sylviadass@usm.my (S.A.D.); venugopal@usm.my (V.B.); 2 Biogenes Technologies, Jalan Maklumat, University Putra Malaysia, Serdang 43400, Malaysia; 3 Centre for Tissue Engineering and Regenerative Medicine (CTERM), Faculty of Medicine, University Kebangsaan Malaysia, Kuala Lumpur 56000, Malaysia; nordinf@ppukm.ukm.edu.my

**Keywords:** latent tuberculosis, heat shock protein 16-kDa, TCR-like antibody, diagnostics, therapeutics

## Abstract

Heat shock protein 16-kDa (HSP 16-kDa) is essential for the survival of *Mycobacterium tuberculosis* (*M. tuberculosis*) during the latent period; hence, a peptide–MHC presentation of HSP 16-kDa could be a potential diagnostic and therapeutic target for latent tuberculosis (LTB). This study aimed to generate a TCR-like single-domain antibody (sDAb)-human IgG1 antibody and subsequently investigate its diagnostic and therapeutic potential in LTB, utilizing a model cell presenting the target peptide. A previously generated TCR-like sDAB that can bind to HSP 16-kDa was first fused to a human IgG1 Fc-receptor via a linker. The fusion product, sDAb-IgG1, was expressed with HEK293-F and was subsequently purified. Its diagnostic potential was investigated via cell-based ELISA utilizing MCF-7 cells peptide-pulsed with HSP 16-kDa peptides. Investigation into the antibody-dependent cell-mediated cytotoxicity (ADCC) of MCF-7 cells was also conducted to investigate its therapeutic potential. Finally, TCR-like sDAb-IgG1 was successfully produced transiently with HEK-293F and was purified using protein A chromatography. The generated antibody was tested using cell-based ELISA, which demonstrated the effective binding of the TCR-like sDAb-IgG1 to the 16-kDa peptide–MHC on the cell surface. The ADCC assay also showed that the antibody effectively mediated the ADCC of MCF-7 cells with the help of 16-kDa peptide–MHC. This allows us to hypothesize the possible utility of the said antibody for both diagnostics and therapeutics of latent tuberculosis after more investigations with clinical samples.

## 1. Introduction

Tuberculosis (TB) is an infectious airborne disease caused by *M. tuberculosis* (MTB) [1]. According to the Global Tuberculosis Report 2023 issued by the World Health Organization (WHO), the number of newly diagnosed TB cases was 7.5 million in 2022. This figure is the highest since the WHO started tracking tuberculosis worldwide in 1995. Globally, 1.30 million people worldwide died of TB in 2022, thus being the second leading cause of death from infectious diseases after SARS-CoV-2 [2]. TB infection can be divided into two main stages. In the initial active stage of a TB infection, the patient’s antibody can destroy the pathogen, but some MTB will escape from the immune system and reside in the alveolar cells of the lungs. This leads to a latency period, where the humoral immunity becomes ineffective and T cells (cellular immunity) are required to initiate the response [3]. Although T cells can fight against MTB infections, the ability of MTB to continuously evade T cell responses makes it difficult to eliminate the pathogen from the host. Moreover, when the host immune system is suppressed or compromised, it will exit the incubation period and reactivate tuberculosis [4]. 

Latent TB is asymptomatic, whereby it does not present the usual clinical symptoms for TB such as fever, cough, expectoration, hemoptysis, or blood in sputum. Currently, there is no gold standard for the diagnosis of latent TB. The most common diagnostic methods used were the tuberculosis skin test (TST) and the interferon-γ release assay (IGRA) to diagnose latent TB [5]. The TST is conducted via the injection of a pure protein derivative (PPD) of TB into the left forearm, and the appearance of circular orange peel-like bumps with a size of 7–8 mm on the skin indicates a positive result [6]. However, the TST has a few major flaws. False positive results can occur in individuals who have received Bacillus Calmette–Guérin vaccines previously. Furthermore, if the body is complicated with malignant tumours, HIV infection, etc., that cause cutaneous anergy, the TST response can be severely reduced and can yield a false negative [7]. On the other hand, IGRA quantitatively detects IFN-γ levels to determine whether the body is infected with MTB. The working principle behind IGRA is the ability of sensitized T lymphocytes to produce IFN-γ during said infection. However, IGRA cannot distinguish between active and latent TB [8]. For treatment, the WHO has recommended multiple drug options for latent tuberculosis infections. The drugs include isoniazid (INH), rifapentine (RPT), and rifampicin (RIF) [9]. However, these drugs often have a high risk of liver toxicity, and patients with liver dysfunction cannot use these drugs. At the same time, MTB is prone to develop drug resistance, making subsequent treatments more difficult [10]. Therefore, it is urgent to find effective diagnostic and treatment methods for latent MTB infections.

The HSP 16-kDa antigen is one of two heat shock proteins produced by MTB, acting as molecular chaperones during protein complex assembly and disassembly [11]. The 16-kDa antigen is primarily expressed by MTB during the stationary phase, in which the bacteria undergo oxygen and nutrient deficiency, highlighting its crucial role in ensuring the survival of MTB during latent infection [12]. In this study, the antigen target peptide sequence is GILTVSVAV, a derivative of MTB HSP16-kDa that can bind to HLA-A2 [13]. The frequency of the HLA-A2 allele is known to be globally common, making the protein a suitable candidate for our study.

In humans, there are four subclasses of immunoglobulin G (IgG), which are IgG1, IgG2, IgG3, and IgG4. Although all subclasses have over 90% identity at the amino acid level, each subclass has different constant regions and unique characteristics in terms of CH2 domains, hinge region length, number of disulphide bonds between chains, and Fc effector functions such as antibody-dependent cell-mediated cytotoxicity (ADCC) and antibody-dependent phagocytosis (ADCP) [14]. When designing monoclonal antibodies for diagnosis or treatment, the isotype and structure of the Fc region not only play an important role in the binding of effector cells, but also influence the binding of antigens [15]. Now, the complete human IgG antibody consists of two major sections—(1) antigen-binding fragments called Fab, and (2) the Fc portion that is responsible for the antibody’s biological activity via binding with Fc gamma receptors (FcγR) on cell surfaces. There are six subtypes of FcγR, which are FcγRI, FcγRIIA, FcγRIIB, FcγRIIC, FcγRIIIA, and FcγRIIIB. Among the four IgG subclasses, IgG1 is the only one that can bind to all FcγR subtypes with the highest affinity and is an effective activator of ADCC and ADCP [16]. Therefore, IgG1 is chosen as the preferred framework for the generation of chimeric TCR-like antibodies.

Due to the potential of 16-kDa HSP as a good antigenic candidate and IgG1 as the framework for chimeric TCR-like antibody design, this study aimed to generate a TCR-like single-domain antibody (sDAb)-human IgG1 antibody against 16-kDa HSP, and subsequently investigate its diagnostic and therapeutic potential for LTB, utilizing a mammalian cell model presenting the target peptide.

## 2. Materials and Methods

### 2.1. Cell Lines

MCF-7 cells and HEK293-F cells were cultured in Dulbecco’s Modified Eagle Medium (DMEM), supplemented with 10% inactivated fetal bovine serum (FBS), 100 U/mL of penicillin, and 100 µg/mL of streptomycin. MCF-7 cells and HEK293-F cells were obtained from Elabscience. All cells were routinely tested as mycoplasma negative.

### 2.2. HSP 16-kDa Peptide and Photolabile Peptide

The HSP 16-kDa peptide and photolabile peptide were purchased from First Base Malaysia, who synthesized it upon order, with a peptide purity of >95%. The amino acid sequence of the HSP 16-kDa peptide in this study was GILTVSVAV, whereas the sequence of the photolabile peptide was KILGFVFJV. The peptides were dissolved in dimethyl sulfoxide (DMSO) diluted in saline at 5 mg/mL and were stored at −80 °C.

### 2.3. Plasmid Construction and Antibody Production

The VH domain gene sequence of the TCR-like sDAb with the ability to bind to the HSP 16-kDa antigen was previously generated in our laboratory [13]. The gene containing human IgG1 heavy-chain constant-chain (UniProtKB: P01857) was synthesized by Integrated DNA Technologies Malaysia and was subsequently cloned onto the pcDNA3.1(+). The plasmid was then transfected into HEK293-F cells (Thermo Fisher Scientific, Waltham, MA, USA) using Invitrogen Lipofectamine (Thermo Fisher Scientific). After that, the antibody was purified from the supernatant using protein A resin (Thermo Fisher Scientific).

### 2.4. SDS-PAGE and Western blot Analysis of Generated Antibody 

Purified antibodies (100 µg) along with a protein ladder were subjected to SDS-PAGE using a 10% resolving and 5% stacking gel under the running condition of 110 V. Then, the gel was subjected to Coomassie brilliant blue staining for 2 h, followed by destaining for 8 h. The gel imaging system was used to scan and save the results.

A Western blot was conducted after SDS-PAGE to identify the expressed antibody. The separated proteins were transferred from the gel to a polyvinylidene fluoride (PVDF) membrane via the semi-dry method at 100 V, 1 A for 1 h. After that, the PVDF membrane was subjected to blocking overnight using 3% BSA in PBS (blocking buffer) at 4 °C. On the next day, the membrane was washed with PBST three times using a shaker for 10 min. Next, rabbit anti-human IgG-HRP (1:1000) diluted with blocking buffer was added and the membrane was incubated at room temperature in the dark for 1 h, followed by washing with PBST on a shaker for 10 min thrice. After washing, the 3,3’-diaminobenzidine(DAB) substrate was added and a gel imaging system was used to scan and save the results.

### 2.5. Generation of HSP 16 kDa Peptide–MHC Complex via Ultraviolet (UV)-Induced Peptide Exchange

The HLA-A2 (heavy-chain) and beta 2-microglobulin (β2-M) (light-chain) vectors were kindly provided by Prof. Dr. Ton Schumacher from the Netherlands Cancer Institute. The generation of a 16-kDa antigen target peptide–MHC complex via UV-induced peptide exchange was performed according to the published protocol [17]. Briefly, the refolded β2-m and HLA-A2 were added to generate refolded HLA-A2 complexes. The refolded HLA-A2 complexes and photolabile peptide were then used to generate photolabile peptide–MHC complexes. Next, upon UV irradiation, the photolabile peptide–MHC complexes were broken, allowing the HSP 16-kDa peptide to replace the photolabile peptide, forming the stable 16-kDa antigen target peptide–MHC complexes.

### 2.6. ELISA Analysis of 16-kDa Antigen Target Peptide–MHC Complexes

This ELISA was conducted to obtain a confirmation on the formation of the 16-kDa antigen target peptide–MHC complexes based on the use of anti-β2M HRP (Abcam, Cambridge, UK) to identify the peptide–MHC complex’s β2M light chain under three distinctive conditions, as follows: (1) photolabile peptide–MHC complexes + HSP 16-kDa peptide+ UV, (2) photolabile peptide–MHC complexes + UV, and (3) photolabile peptide–MHC complexes. Each sample was conducted in triplicate. First, 10 μL streptavidin (2 μg/mL in PBS) was added to each well, followed by incubation for 2 h at 5 °C. Each well was washed three times with 300 mL PBST, and was then blocked with 300 μL 2% BSA for 1 h at 5 °C. After that, each well was washed three times with 300 mL PBST. Next, the samples were added into their respective wells and incubated at 5 °C for 1 h, followed by washing with PBST thrice. The anti-β2M HRP (100 μL, 1:5000) was then placed in the well and was incubated at 5 °C for 1 h, followed by washing with PBST three times. Lastly, 100 μL ABTS was added to each well and was incubated at 37 °C for 10 min. The OD at 408 nm was subsequently read using a microplate reader. 

### 2.7. ELISA Analysis of TCR-like Antibody (A2-IgG1) Binding to 16-kDa Antigen Target Peptide–MHC Complexes 

This ELISA was conducted to evaluate the TCR-like antibody (A2-IgG1) binding to 16-kDa antigen target peptide–MHC complexes under three distinctive conditions, as follows: (1) 16-kDa antigen target peptide–MHC complexes, (2) photolabile peptide–MHC complexes, and (3) PBS only. Each sample was conducted in triplicate. Firstly, each well was coated with 100 μL of the sample (2 μg/mL in PBS) for 2 h at room temperature, followed by washing three times with 300 mL PBST. After that, 100 μL of TCR-like antibody (2 μg/mL in 2% BSA) was added and was incubated for 6 h, followed by washing three times with 300 mL PBST. Next, 100 μL of rabbit anti-human IgG-HRP (1:5000, Invitrogen) was added to the wells and was incubated for 1 h at room temperature, followed by washing three times with 300 mL PBST. Lastly, 100 μL ABTS was added to each well and was incubated at 37 °C for 10 min. The OD at 408 nm was subsequently read using a microplate reader.

### 2.8. Human Peripheral Blood Mononuclear Cells (PBMCs) Isolation and Handling

PBMCs from healthy volunteer donors were isolated from heparinized peripheral blood using density gradient centrifugation with the help of lymphocyte separation medium (MP Biomedicals, Santa Ana, CA, USA). After isolation, PBMCs were resuspended in RPMI 1640 medium supplemented with 10% FBS and 1% penicillin/streptomycin solution. The PBMCs were then counted using the trypan blue exclusion assay; they were subsequently resuspended in cold freezing medium (FBS with 10% DMSO (Sigma-Aldrich, Munich, Germany)) and were stored as 4 × 10^6^ cells/vial at −80 °C until further use.

### 2.9. Peptide Pulsing of MCF-7 Cells with 16-kDa HSP Peptides

MCF-7 cells (3 × 10^6^, 25 mL) were plated in 150 mm Petri dishes overnight. The following day, the media were supplemented with IFN-γ (Thermo Fisher Scientific) at a concentration of 250 U/mL^17^. After 48 h of incubation, MCF-7 cells were co-incubated with HSP 16-kDa peptides at 37 °C for 3 h to enable the cells to present the antigen on its surface.

### 2.10. Cell-Based ELISA

Peptide-pulsed MCF-7 cells were seeded in a 24-well plate at a density of 1 × 10^5^ cells/mL, and were incubated at 37 °C, 5% CO_2_. Upon confirming the adherence of the cells, 2 mL of PBS was used for each well to wash the cells. Next, the TCR-like A2-IgG1 antibody was added to each well, achieving a final concentration of 10 µg/mL, and was incubated for 1 h (37 °C, 5% CO_2_). The cells were washed with PBS, followed by incubation with 100 µL of anti-human IgG HRP (1:1500, Invitrogen) for 45 min. Finally, 400 µL of ABTS was added to each well and was incubated for 30 min. The absorbance value was measured at a wavelength of 408 nm.

### 2.11. ADCC Assay

The concentration of PBMCs (effector cells) and peptide-pulsed MCF-7 cells (target cells) used in this assay was 1 × 10^6^/mL, respectively. First, 100 mL of target cells were seeded in 96-well plates, followed by the addition of 400 mL of effector cells. After that, A2-IgG1 antibody was added to the co-culture, achieving a final concentration of 10 µg/mL, and was incubated for 8 h in an incubator at 37 °C, 5% CO_2_. The viability of MCF-7 cells was detected using the MTT assay at a wavelength of 490 nm. The formula for calculating cell viability is as follows:

$$Cell\;Viability\;\left(\%\right)=\frac{Mean\;{OD}_{sample}}{Mean\;{OD}_{blank}}\times 100$$

### 2.12. Statistical Analyses

The experimental data were analyzed via Student’s *t*-test; differences with a *p* < 0.05 (*) were considered significant. Results were denoted as mean ± standard error (SE). All graphs and statistical analyses were performed and generated using GraphPad Prism 7.

## 3. Results

### 3.1. Expression and Purification of TCR-like Single-Domain Antibodies

**Figure 1:**
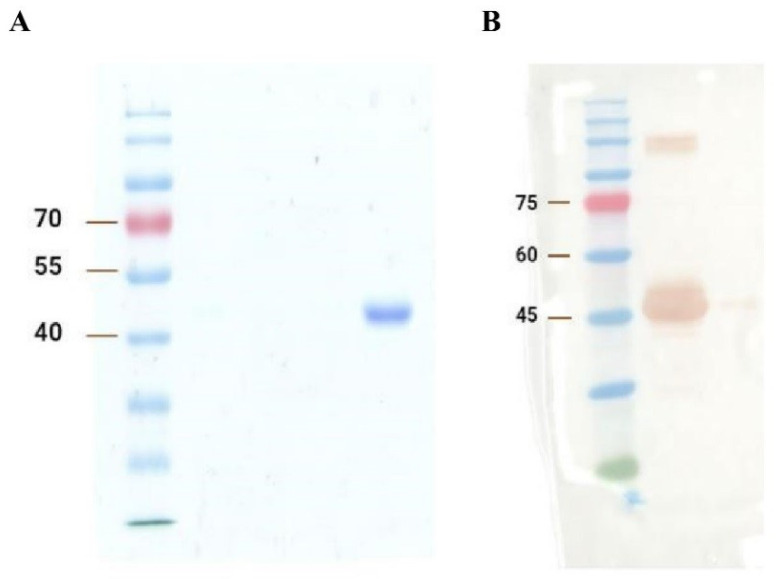
(**A**) SDS-PAGE and (**B**) Western blot analysis of the generated TCR-like domain antibody (A2-IgG1). The first gel was dyed with Coomassie brilliant blue and destained overnight for SDS-PAGE analysis, whereas the second gel was transferred to the PVDF membrane for the Western blot detection of A2-IgG1.

The SDS-PAGE and Western blot results are shown in Figure 1A and 1B, respectively. Generally, based on Figure 1, the results indicate the satisfactory expression and purification of A2-IgG1. The expected size of domain antibodies (~42 kDa) was obtained in both analyses. Western blot further confirms that the ~42 kDa band was the generated soluble domain antibody. High concentration samples lead to protein aggregation. These aggregates may not fully dissociate during SDS treatment, resulting in abnormal migration during electrophoresis and the formation of an extra band above 75 kDa on the Western blot in Figure 1B.

### 3.2. Detection of 16-kDa Antigen Target Peptide–MHC Complexes Formation using ELISA

**Figure 2:**
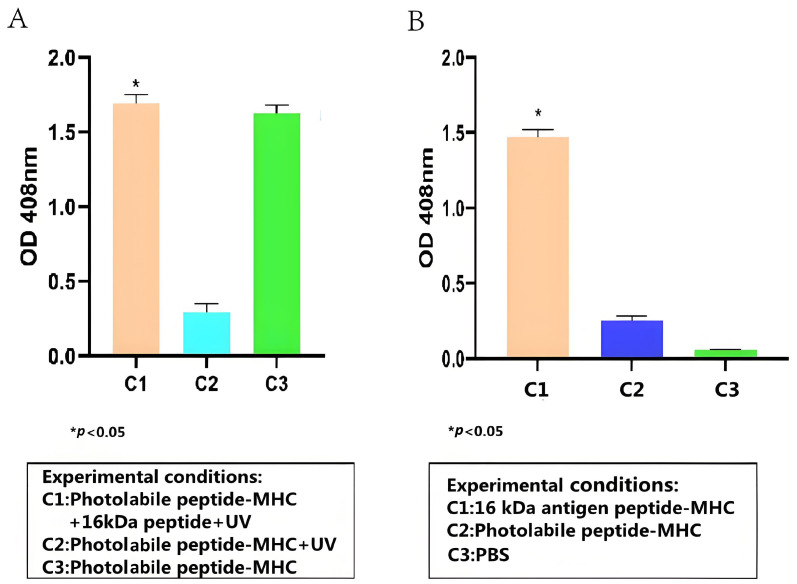
ELISA test results for (**A**) detection of 16-kDa antigen target peptide–MHC complex formation (Student *t*-test, n = 3, differences with a *p* < 0.05 (*) were considered significant). (**B**) Detection of 16-kDa antigen target peptide–MHC complexes using TCR-like sDAb (A2-IgG1) (Student *t*-test, n = 3, differences with a *p* < 0.05 (*) were considered significant). TCR-like sDAb (A2-IgG1) with a concentration of 10 µg/mL were added to each group, and the ELISA readings indicated the TCR-like sDAb (A2-IgG1) significantly recognizes 16-kDa antigen target peptide–MHC complexes.

To test whether the HSP 16-kDa peptide can bind stably to HLA-A2, peptide–MHC stability ELISA was conducted, and its results are presented in Figure 2A. Overall, the stability ELISA demonstrated that the HSP 16-kDa peptide can replace the photolabile peptides that are conjugated to HLA-A2, forming stable peptide–MHC complexes. Based on Figure 2A, the high readout from C1 and C3 showed that the anti-β2-M HRP antibody successfully binds to the 16-kDa antigen target peptide portion and the photolabile peptide portion of the peptide–MHC complex, respectively. The low reading of C2 indicated that the UV-sensitive photolabile peptide was broken down upon UV irradiation, thus becoming unstable in the absence of a peptide substitute, causing its degradation and the anti-β2-M HRP antibody could not bind to it. Because the photolabile peptide–MHC complex was not exposed to UV for C3, a high readout value was achieved.

### 3.3. TCR-like sDAb (A2-IgG1) Binding to 16-kDa Antigen Target Peptide–MHC Complexes 

The binding capacity between the TCR-like sDAb (A2-IgG1) and the 16-kDa-HLA-A2 complex was evaluated via cell-based ELISA. Overall, based on Figure 2B, the TCR-like sDAb (A2-IgG1) binds significantly to the peptide–MHC complexes. Compared to photolabile peptide–MHC complexes and PBS only, the binding between A2-IgG1 and HSP 16-kDa is significantly higher.

### 3.4. Detection of HSP 16-kDa Peptide Presentation in Cell Lines

**Figure 3:**
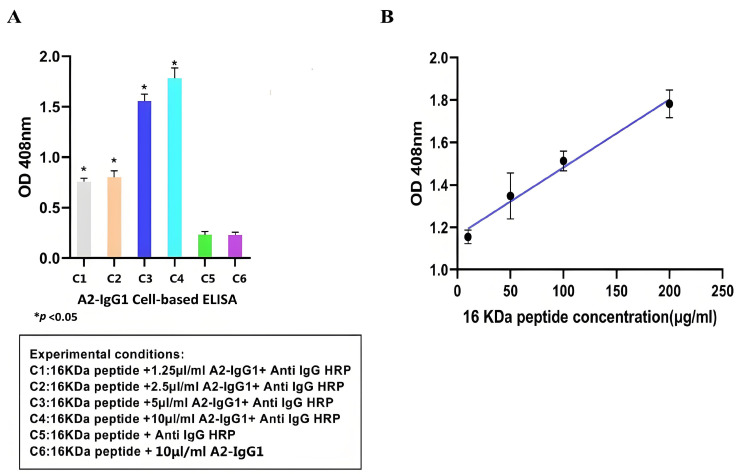
(**A**) Detection of 1 16-kDa antigen target peptide–MHC complexes on the MCF-7 cell surface using TCR-like sDAb (A2-IgG1) at different concentrations. The high reading of the cell-based ELISA indicates that the antibody can effectively recognize antigenic peptides on the surface of cells. (**B**) Standard curves were drawn using cell-based ELISA to detect HSP 16-kDa peptide loaded at different concentrations onto the cell surface. The regression equation for the HSP 16-kDa peptides is y=0.003211x+1.160 (Student’s *t*-test, n = 3, differences with a *p* < 0.05 (*) were considered significant).

Cell-based ELISA is an immunoassay method based on the high specificity of antigen–antibody binding and the efficient catalytic activity of enzymes. It has been widely used in clinical medicine, as well as in the animal and plant fields [18]. After demonstrating the specific affinity of antibodies for soluble recombinant 16-kDa-MHC molecules, we used cell-based ELISA to test whether antibodies recognize the peptide–MHC complexes displayed on the surface of peptide-pulsed MCF-7 cells. Overall, our cell-based ELISA demonstrated that the generated TCR-like sDAb (A2-IgG1) enhances ADCC activity against MCF-7 cells that were peptide-pulsed with the 16-kDa antigen target peptide. Based on Figure 3A, the higher absorbance value indicated that TCR-like sDAb (A2-IgG1) can effectively detect 16-kDa peptide–MHC on MCF-7 cell lines. As the concentration of added antibodies increases, the absorbance value also increases significantly, showing a similar antibody concentration-dependent binding trend (Figure 3A).

At the same time, the absorbance values of 16-kDa peptides at different concentrations (10 µg/mL, 50 µg/mL, 100 µg/mL, and 200 µg/mL) were detected using A2-IgG1 (10 µg/mL). Standard curves for antibodies of different concentrations are shown (Figure 3B). 

### 3.5. Antibody-Dependent Cellular Cytotoxicity(ADCC) of MCF-7 Cells Expressing the HLA-A2 Gene Mediated by a TCR-like sDAb (A2-IgG1)

**Figure 4:**
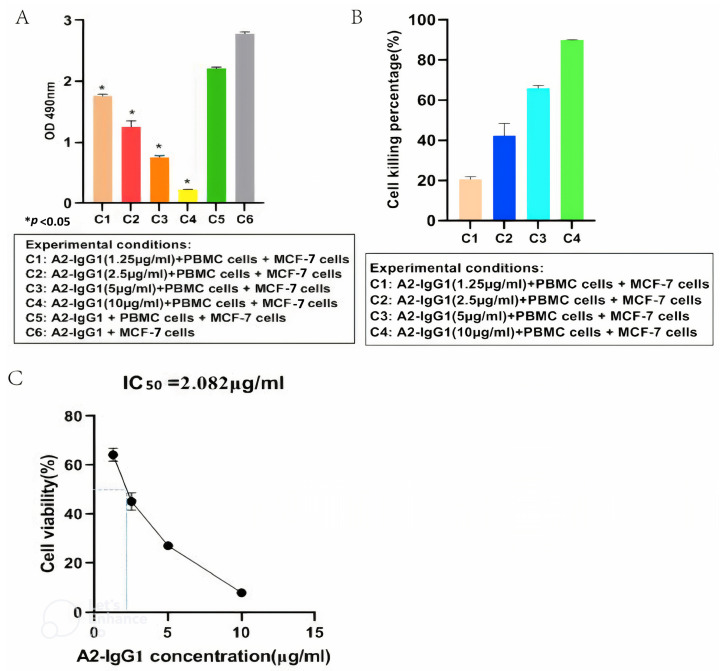
(**A**) MTT assay for detecting ADCC in MCF-7 cells mediated by different concentrations of TCR-like sDAb (A2-IgG1). (**B**) Cell viability rates correspond to different absorbance values. This indicates that our A2-IgG1 antibody can significantly induce ADCC in MCF-7 cells expressing the HLA-A2 gene. (**C**) Calculation of the antibody IC_50_ = 2.082 µg/mL (Student’s *t*-test, n = 3, differences with a *p* < 0.05 (*) were considered significant).

An ADCC assay was performed to determine the ability of the generated A2-IgG1 antibody to induce ADCC in MCF-7 cells expressing HLA-A2. For the quantification of ADCC, PBMCs were used as effector cells, and four different concentrations (1.25 µg/mL, 2.5 µg/mL, 5 µg/mL, and 10 µg/mL) of TCR-like antibody (A2-IgG1) were used, respectively. After 100 µg/mL of antigenic peptide was loaded into MCF-7 cells, 10^5^ cells/well (triplicates) were inoculated into 96-well plates. Target cells and effector cells were incubated at a ratio of 1:4. Different concentrations of TCR-like antibodies (A2-IgG1) were added. At the same time, two blank control groups (only target cells; target cells and effector cells) were set. After 8 h, an ADCC assay was conducted using MTT and the absorbance values of each group were measured at a wavelength of 490 nm using an enzyme-linked immunosorbent assay (Figure 4A). As shown in Figure 4B, the A2-IgG1 antibody showed significant cytotoxicity in MCF-7 cells forming 16-kDa antigen target peptide–MHC complexes, and as the concentration of antibodies increased, the cell killing percentage significantly increased. Finally, the antibody’s IC_50_ value of 2.082 µg/mL was determined (Figure 4C). This indicates that the A2-IgG1 antibody can significantly induce ADCC in MCF-7 cells expressing HLA-A2.

## 4. Discussion

The immune surveillance of the intracellular proteome of all nucleated cells is carried out by the major histocompatibility complex (MHC) class I system, commonly known as the human leukocyte antigen (HLA) system [19]. Antigen peptide presentation distinguishes between malignant or infected cells and their healthy counterparts, resulting in the creation of abnormal cells that serve as the basis for identification [20]. Therefore, the 16-kDa peptide MHC can give precise diagnostic and therapeutic targets for latent MTB infection. TCR-like sDAb recognizes HSP 16-kDa peptides that bind to MHC molecules, allowing them to target such inaccessible antigens. It binds to MHC-presenting peptides found on the surface of target cells. Therefore, the TCR-like sDAb-IgG1 shows a promising theoretical basis for clinical applications in the diagnosis and treatment of cellular tuberculosis infection.

IgG1 is the most abundant IgG subclass in human serum and is important for mediating antibody responses against pathogens. It achieves this by combining its variable domain with soluble proteins and membrane protein antigens, while simultaneously activating the innate immune system. IgG1 can effectively bind to C1q, leading to complement-dependent cytotoxicity (CDC), and can bind to each different Fc receptor, leading to antibody-dependent cell-mediated cytotoxicity (ADCC). Historically, the IgG1 subclass has been the preferred method for designing therapeutic and diagnostic antibodies [21]. It has ideal biophysical properties, including a relatively high thermal stability, monomer properties, and an average flexible hinge region containing only two disulphide bonds. Therefore, we used the IgG1 framework to express chimeric TCR-like antibodies that can recognize the 16-kDa peptide–MHC, and successfully expressed and purified them. 

The cell-based ELISA is particularly popular in diagnostic testing, as it can be applied to detect infectious pathogens themselves and the antibody reactions they cause in the host; therefore, cell-based ELISA is useful in identifying infected individuals who may spread the disease or require treatment, as well as in identifying rehabilitation individuals who have previously been infected in epidemiological monitoring [22]. Some chronic infections, especially when the load of infectious sources decreases, may be difficult to detect if solely relying on detecting the source of infection. However, continuous antibody reactions can be detected in these individuals, and cell-based ELISA helps to accurately locate these individuals [23]. The limit of detection (LOD) and limit of quantification (LOQ) are important quality factors to determine the quality of an immunoassay. The presence of any detectable signal from the particular instrumental setup that can be attributed to the target being studied is what is referred to as LOD; LOQ is the threshold at which measurements are precise enough for quantitative analysis [24]. The regression equation for the HSP 16-kDa peptides is as follows: *y* = 0.003211*x* + 1.160. The correlation coefficient values are as follows: δ = 0.003211, S = 0.022903, R^2^ = 0.9272, LOD = 3δ/S = 0.421 µg/mL, and LOQ = 10δ/S = 1.402 µg/mL. The cell-based ELISA results indicate that our TCR-like sDAb-IgG1 can effectively recognize the 16-kDa peptide–MHC on the cell surface. This shows that our TCR-like sDAb-IgG1 has good potential in diagnosing specific antigen complexes of latent tuberculosis infection. In terms of antigen specificity, we utilized a non-specific, photolabile peptide as a negative control (Figure 2). This is critical because the target antigen is an MHC Class I molecule presenting a 9-mer peptide. As a control, the 9-mer peptide has been replaced with a photolabile peptide to avoid non-specific binding towards the MHC class I molecule but specifically for the MHC class I molecule presenting the peptide of interest. Through these control experiments, we confirmed the specificity of the TCR-like antibodies expressed, and ensured the reliability of our experimental results.

ADCC is an adaptive immune response mainly composed of NK cells passing through CD16A (FcγRIIIA) receptor-mediated binding to the Fc portion of IgG antibodies, triggering target cell lysis [25]. PBMCs include lymphocytes (T cells, B cells, and NK cells), monocytes, and dendritic cells. In humans, the frequencies of these populations vary between individuals, but typically lymphocytes are in the range of 70–90% [26]. The NK cell subset expressing CD16A in PBMCs is the ideal effector cell in the ADCC test [27]. The experimental results indicate that our TCR-like antibody can effectively mediate the ADCC of MCF-7 cells with 16-kDa peptide MHC. These data show that the TCR-like antibody we produced has a good potential for application in the treatment of latent tuberculosis infection.

In our research, we mainly focus on the preliminary screening and functional validation of antibodies, aiming to demonstrate their efficacy and specificity in an in vitro model. We realize that this is only the initial stage of antibody development and subsequent research requires more comprehensive and in-depth functional testing, including in vivo animal model research and long-term safety assessments.

## 5. Conclusions

The results from this preliminary study have indicated that we have successfully demonstrated a novel TCR-like single-domain antibody—human IgG1 antibody—which can effectively bind to the 16 kDa HSP–MHC complex and induce ADCC activity. This study serves as a concept validation platform, demonstrating the potential of the antibody in recognizing and responding to specific antigen complexes of latent *M. tuberculosis*. However, our experiment did not directly evaluate the application of this antibody in the treatment or diagnosis of latent tuberculosis. Therefore, the effectiveness of the antibody against latent tuberculosis would require further experimentation.

For future studies, animal models can be used to study the processes that occur during the infection process of various animal species’ diseases. These models will help to better understand the immune responses produced by animals, study the mechanisms of diseases, and test the therapeutic effects of emerging drugs [28]. Establishing an animal model using mice that can express HLA-A2 on the cell surface [29] infected with *M. tuberculosis* H37Rv [30] would be beneficial for the validation of the effectiveness of our antibodies in the diagnosis and treatment of latent *M. tuberculosis* infection. Due to the differences in the constant regions of the IgG1, IgG2, and IgG4 subclasses of IgG, there are significant differences in their functions [16]. Therefore, further investigation of the different antibody isotypes against latent tuberculosis infection will further enhance the efficacy of latent tuberculosis treatment. 

## Data Availability

The datasets generated during and/or analyzed during the current study are available from the corresponding author upon reasonable request.
